# Network Receptive Field Modeling Reveals Extensive Integration and Multi-feature Selectivity in Auditory Cortical Neurons

**DOI:** 10.1371/journal.pcbi.1005113

**Published:** 2016-11-11

**Authors:** Nicol S. Harper, Oliver Schoppe, Ben D. B. Willmore, Zhanfeng Cui, Jan W. H. Schnupp, Andrew J. King

**Affiliations:** 1 Dept. of Physiology, Anatomy and Genetics (DPAG), Sherrington Building, University of Oxford, United Kingdom; 2 Institute of Biomedical Engineering, Department of Engineering Science, Old Road Campus Research Building, University of Oxford, Headington, United Kingdom; 3 Bio-Inspired Information Processing, Technische Universität München, Germany; 4 Department of Biomedical Science, City University of Hong Kong, Kowloon Tong, Hong Kong; Hebrew University of Jerusalem, ISRAEL

## Abstract

Cortical sensory neurons are commonly characterized using the receptive field, the linear dependence of their response on the stimulus. In primary auditory cortex neurons can be characterized by their spectrotemporal receptive fields, the spectral and temporal features of a sound that linearly drive a neuron. However, receptive fields do not capture the fact that the response of a cortical neuron results from the complex nonlinear network in which it is embedded. By fitting a nonlinear feedforward network model (a network receptive field) to cortical responses to natural sounds, we reveal that primary auditory cortical neurons are sensitive over a substantially larger spectrotemporal domain than is seen in their standard spectrotemporal receptive fields. Furthermore, the network receptive field, a parsimonious network consisting of 1–7 sub-receptive fields that interact nonlinearly, consistently better predicts neural responses to auditory stimuli than the standard receptive fields. The network receptive field reveals separate excitatory and inhibitory sub-fields with different nonlinear properties, and interaction of the sub-fields gives rise to important operations such as gain control and conjunctive feature detection. The conjunctive effects, where neurons respond only if several specific features are present together, enable increased selectivity for particular complex spectrotemporal structures, and may constitute an important stage in sound recognition. In conclusion, we demonstrate that fitting auditory cortical neural responses with feedforward network models expands on simple linear receptive field models in a manner that yields substantially improved predictive power and reveals key nonlinear aspects of cortical processing, while remaining easy to interpret in a physiological context.

## Author Summary

Linear filter descriptions of sensory neurons have been with us since the 1970s, and have been enormously influential. But such models, and more recent nonlinear variants, are rather like modeling the entire network as a single neuron, failing to account for the neuron's response being a consequence of a network of many nonlinear units. Here we show how these limitations can be overcome by using recent advances in machine learning to fit “network receptive field models” to neural responses to natural sounds. Feedforward networks of 1–7 nonlinearly-interacting lower-order model neurons are required to model a cortical receptive field. Each lower order neuron is tuned to somewhat different stimulus features, arranged together in complex but interpretable structures, which cover a far wider range of sound frequencies and delays than current receptive field models indicate. The NRF models capture important nonlinear functional characteristics in auditory cortical neurons, including multiplicative gain control and conjunctive feature selectivity, where neurons respond when certain features are present together but not in isolation. This enables NRFs to predict the responses of auditory cortical neurons with considerably greater accuracy than conventional models.

## Introduction

Developing models capable of quantitatively predicting neural responses to sensory stimuli is key to understanding the neural computations underlying perception. A widespread model of sensory neurons, including cortical sensory neurons, is the receptive field (RF), which describes the best-fitting linear transformation from the stimulus to the neural response [1–16]. RF models, although simple and useful, are only moderately effective in capturing neural responses since processing by networks of neurons includes highly nonlinear operations. Consequently, they can fail to produce adequate descriptions of neural responses, particularly to natural stimuli [17,18].

While spectrotemporal receptive fields (STRF) of neurons in primary auditory cortex (A1) can be quite broad and complex, many of them are punctate, typically little more than a point in time and frequency, indicating little of the likely complexity of cortical processing [19] (although see [12]). Adding specific nonlinearities to STRF models [17], for example by applying output nonlinearities [19,20] to create linear-nonlinear (LN) models, improves prediction somewhat. However, basic LN models, consisting of just a single STRF and an output nonlinearity, still fail to capture the interactions of sensory filters that are bound to occur naturally in the neural networks of ascending sensory pathways. Recently, more complex and often nonlinear STRF models [20–25] of A1 neurons have achieved improved predictions of experimental data, although sometimes at the expense of being very computationally intensive. These newer models have tended to concentrate on better modeling of features local to the neuron, such as synaptic depression [23] or refractoriness [22]. Other valuable approaches adopted to characterize the feature selectivity of A1 neurons are more phenomenological in nature [20].

Here we take a very different approach, one that embodies the fact that a neuron's response is the result of it being embedded in a network of many neurons, each of which is a nonlinear unit. We take advantage of recent advances in the training of artificial neural networks [26] to produce a new type of RF model, the network receptive field (NRF), which can be rapidly fitted to neural response data. The NRF model is composed of a hierarchical feedforward network of 20 LN units, embodying the fact that cortical neurons integrate the output of many lower order neurons. Although our choice of 20 possible feed-forward connections does not reflect the full range of converging inputs that cortical neurons receive, this approach stands in contrast to the above mentioned recent models of A1 responses [20–24], which use only one, or in some cases two, STRF-like units. In fact, we show here that up to seven effective units are required to model a cortical receptive field.

Receptive field models tend to include large numbers of free parameters, which can lead to problems with “overfitting”: the many free parameters of the model may capture unimportant or coincidental details or noise in the training set. This can result in the model appearing to successfully capture the stimulus-response relationships in the training set, but subsequently performing poorly when the model is used to predict neural responses to novel stimuli that were not part of the training set. To prevent the risk of overfitting affecting our results we took the following steps: First, during model fitting, the NRF was regularized by the summed magnitudes of the network's weights (*L*_1_-norm), which automatically prunes away superfluous weights and hidden units (from an initial 20 hidden units). This produces parsimonious and readily interpretable connection patterns that provide insights into the underlying circuitry. Second, we made extensive use of cross-validation during model training (see below) and assessed the performance of all models using a generalization test set which the models had not been exposed to during training.

Together, the regularization, cross-validation and generalization testing adopted here ensure that the improved performance exhibited by our NRF models is not a trivial consequence of the larger number of degrees of freedom that these models can bring to bear, but rather reflect the fact that the structure of these models renders them better able than conventional LN receptive field models to capture aspects of the sensory processing performed by the auditory pathway. Thus, using electrophysiological recordings from ferret auditory cortex, we find that NRF models consistently outperform LN models in predicting the responses of auditory cortical neurons to natural stimuli. The fitted NRF models of auditory cortical neurons reveal sensitivity over substantially wider time and frequency ranges than conventional LN and STRF models, and the NRFs also reveal distinct nonlinear properties, including gain control and conjunctive feature selectivity, features that may be critical to auditory cortex function. Conjunctive feature selectivity, where neurons respond when certain features are present together but not in isolation, allows neurons to show increased selectivity to specific complex spectrotemporal structures and may provide a valuable stage in the sound recognition process.

## Results

### Network receptive field models of neural responses in auditory cortex

**Fig 1:**
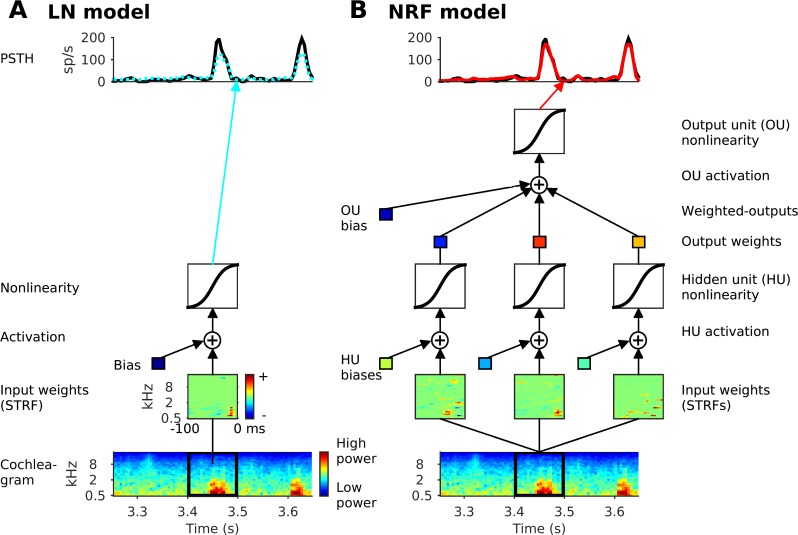
Schematics of the models. (A) The linear-nonlinear (LN) model. (B) The network receptive field (NRF) model, a feedforward neural network

To investigate the ability of NRFs to account for cortical sensory responses, we fitted models to neural responses to clips of natural sounds. Seventy-six single-unit responses were recorded with multi-channel electrodes in the ferret primary auditory cortical areas, A1, and the anterior auditory field (AAF). The stimuli comprised 20 clips of natural acoustic scenes, each of 5 s duration, including ferret vocalizations, speech, and environmental sounds. The model fitting process is shown schematically in Fig 1. and described in detail in the Materials and Methods. The first step in the NRF model was to generate a first order approximation of auditory nerve response patterns to the stimuli, referred to here as the “cochleagram”, by measuring the log amplitude of the sound in each of 34 log-spaced frequency channels, spanning 0.5 to 22.6 kHz with ⅙ octave spectral resolution and 5 ms temporal resolution. The task of the model was then to predict the firing patterns recorded from the cortical neurons, also binned with 5 ms time resolution, from the previous 100 ms (20 time bins) of stimulus history.

In accordance with principles of model selection and assessment [27], we divided the neural response data into a cross-validation set and a test set. The cross-validation set was then divided again into a training set that was used to fit the model parameters, and a validation set on with which the model's capacity to predict neural response was then assessed. By this means, the optimum value for general settings of the model (hyperparameters, such as the degree of regularization) could be determined. This fitting was repeated ten times for ten different ways of dividing the cross-validation set, to ensure a robust assessment of the optimum model hyperparameter values. Note that the model fits, for both the LN and NRF models, tended to differ little in their receptive field forms over these ten fits, despite having slightly different datasets and different randomly chosen weight initializations, indicating the robustness of the fitting procedure (for details and quantification see Materials and Methods). Once the optimum hyperparameters were obtained, the model was re-fitted using the full cross-validation dataset. Finally, the test dataset that was put aside was used to assess the fitted model's capacity to predict responses to sounds not encountered at any stage of the fitting (i.e. to “generalize”). All model performance data reported below refer to results obtained with the test set.

A conventional LN model and an NRF model were fitted for each cortical neuron in our dataset. The LN model comprised a linear STRF, used to calculate the activation of the model neuron, and a sigmoidal output nonlinearity (Fig 1A). The linear STRF on its own also provided a basic linear (L) model. The NRF model was a rate-based, feed-forward neural network (a multilayer perceptron), with units that integrate inputs linearly followed by a nonlinear transformation to produce their output [28]. The NRF effectively computes a weighted sum of several LN models, where each hidden unit (HU) instantiates one LN model, and their outputs are combined linearly as they converge on the output unit (OU). The resulting OU activation passes through a further sigmoidal nonlinear activation function (Fig 1B) to yield the NRF model’s prediction of the neural firing rate. The network units have no memory from time point to time point; the model does not use any recurrent or convolutional elements. All models (LN and NRF) were fitted by minimizing the squared error between the model’s estimate of the neural response and the actual neural firing rate (see Materials and Methods for details). Importantly, *L*_1_-norm regularization of the connection weights was used to find a parsimonious representation. A recently developed algorithm [26] allowed for good NRF models to be fitted rapidly and efficiently for all 76 cortical neurons in our dataset.

### NRF models describe neural responses better than LN models

**Fig 2:**
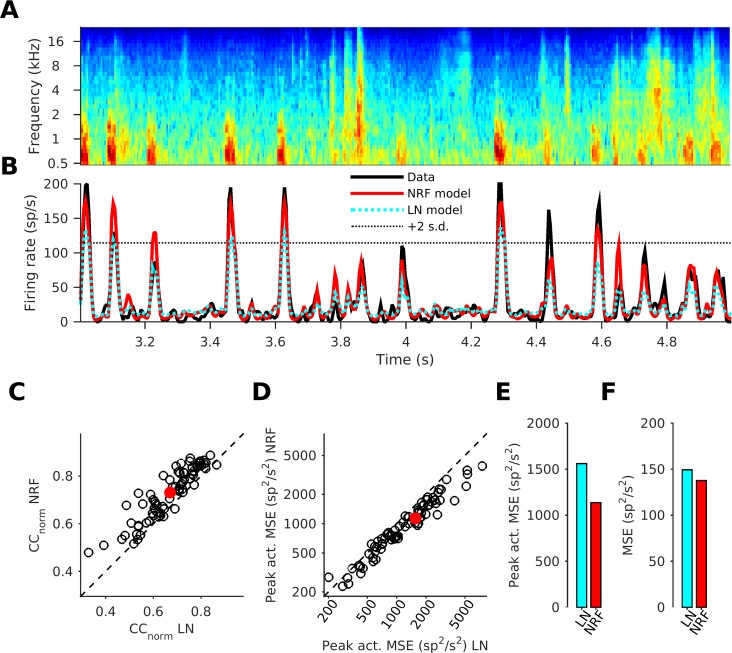
A neural network receptive field model predicts the response of auditory cortical neurons better than the LN model. (A) Cochleagram for a 2 s sound stimulus snippet. (B) The neural response firing rate to the stimulus snippet shown in A (black line) for one example neuron, shown alongside the predicted responses of the LN-model (dotted cyan line) and the NRF model (red line). The thin dotted black line indicates the 2*σ*-threshold, which was used to identify periods of large response peaks. (C) Prediction quality (normalized correlation coefficient) of the NRF model plotted against that of the LN-model for all 76 neurons in our dataset. (D) Mean squared error (MSE) of the prediction during peak response times of the NRF models plotted against error of the LN-models. (E) Average peak activity MSE (pMSE) over the whole dataset for the NRF and the LN models. (F) Average MSE of the predictions generated by NRF and LN models as in E, but calculated across the whole response to the test stimuli, not just the peak response period.

To assess the models’ predictive power, we measured how well they were able to predict responses to a “test set” of stimuli which were not part of the training set used during model fitting. The NRF tends to better predict the amplitude of sharp peaks in the observed neural response than the LN model (Fig 2A and 2B, seconds 3–4 are from the training set, seconds 4–5 are from the test set). We quantified the quality of the response prediction by calculating the normalized correlation coefficient (*CC_norm_*) between predicted and observed neural responses [29,30]. A *CC_norm_* of 0 would indicate that the model fails to predict the neural responses any better than chance, while *CC_norm_* values of 1 indicate predictions that are at the highest achievable accuracy (see Materials and Methods). For the great majority of neurons (70/76), the NRF achieved higher *CC_norm_* values than the LN model (p = 6.3×10^−15^, n = 76, sign test; Fig 2C), with the mean *CC_norm_* for the NRFs being 0.73, compared to 0.67 for the LN model. The *CC_norm_* for the L-model, the prediction using the STRF but without processing through the fitted sigmoidal output nonlinearity, was 0.60, significantly less than both the NRF (76/76, p = 2.6×10^-23^, n = 76, sign test) and the LN model (75/76, p = 2.0×10^−21^, n = 76, sign test). The *CC_norm_* value for the NRF model may approach the maximum possible given the duration of the STRFs (100 ms) used by the NRF model [31] (See Discussion). We also report the raw mean correlation coefficient (*CC*_*raw*_), which was 0.61 for the NRF model, and 0.56 for the LN model, to enable comparison with previous publications (but note, that differences in raw *CC*_*raw*_ values between different studies are difficult to interpret, as will be discussed further below). As expected, for this measure too, the great majority of neurons (70/76) were significantly better fit by the NRF than the LN model (p = 6.3×10^−15^, n = 76, sign test). The *CC*_*raw*_ for the linear model was 0.50, significantly less than both the NRF (76/76, p = 2.6×10^−23^, n = 76, sign test) and the LN model (75/76, p = 2.0×10^−21^, n = 76, sign test).

The capacity of the NRF model to predict better than the LN model is also robust to the exact choice of test set. This is evident from examining the prediction quality for the validation sets, which, in order to require the model to generalize across stimulus types, comprised 2 of the 20 sounds, chosen at random. The mean *CC_norm_* for the validation set, averaged over all 10 folds, is greater for the NRF model (0.76) than the LN model (0.71), with significantly more neurons (69/76) showing a greater *CC_norm_* for the NRF model than the LN model (p = 6.4x10^-14^, n = 76, sign test).

To investigate how well the models are able to predict peak responses in the test set, we also measured the “peak activity mean squared error”, which was defined as the MSE between the observed firing rates and those predicted by the models during periods where the observed firing rate exceeded two standard deviations above the mean firing rate (the “2σ-threshold”, dotted line in Fig 2B). It is readily apparent that the peak activity MSE of the NRF model is smaller than that of the LN model for the great majority of neurons (p = 5.2×10^-16^, n = 76, sign test; Fig 2D). The peak activity MSE, averaged over all neurons, was 27% smaller for the NRF model than for the LN model (Fig 2E). This reduction of prediction error during periods of peak excitation appears to drive the improved performance of the NRF model relative to the LN model. This is indicated by much smaller average improvement (8%) for the NRF over the LN model when the MSE was measured over the entire neural response (Fig 2F).

Note that all the model performance data in Fig 2. were calculated exclusively from test sets that the models were not exposed to during training. This is essential to ensure that appropriate model comparisons were made. NRF models have significantly more free parameters than conventional LN models, and, if tested on the training data, might trivially outperform the LN models by overfitting noise in the training data, but such overfitting would become disadvantageous when the models were used to make predictions for novel stimulus sets. The fact that NRF models outperform LN models on previously unseen data indicates that the NRF models mimic aspects of the behavior of the cortical neurons which the structure of LN models cannot account for.

### NRFs reveal that cortical neurons are better described by the interaction of multiple, diverse sub-receptive-fields

**Fig 3:**
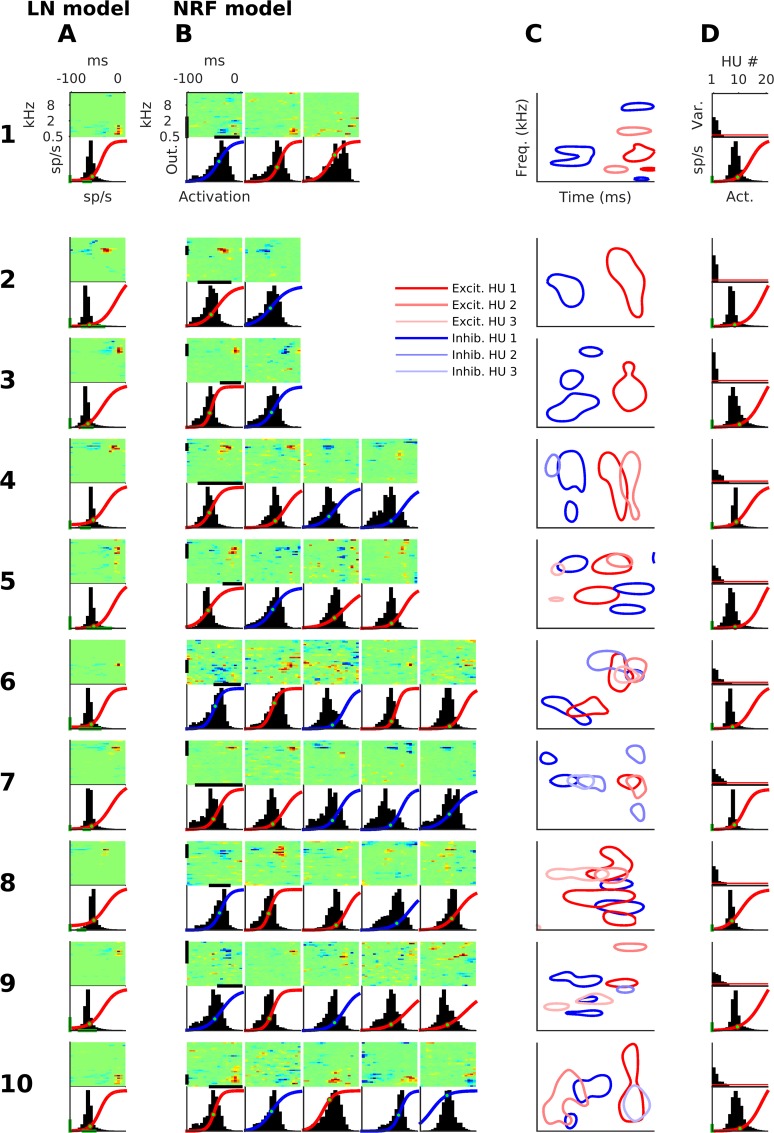
Example STRFs and nonlinearities for both models. Each numbered row is an example neuron. (A) STRFs (top) and nonlinearities (bottom) for the LN model. Green bars mark 0–20 sp/s. The nonlinearities are superimposed over the distribution of activations. The green dot on the nonlinear activation function marks the mean output value. (B) Hidden unit (HU) 'STRFs' (top) and nonlinearities (bottom) for the NRF model. If the nonlinearity curve is red, it is an excitatory HU, if blue, it is inhibitory. Otherwise format as in A. Note that the STRFs of inhibitory units have been multiplied by -1 for display purposes, to indicate the direction of their influence on the final neural output (See Materials and Methods, The displayed STRFs). One would therefore not necessarily expect to observe extensive inhibitory areas such as those in some of these HU display STRFs in physiological recordings, as such inhibitory fields would most likely manifest in biological networks as excitatory fields that feed on to the next neuron via inhibitory synapses. (C) HU STRFs in B plotted together as contours. The contours are at 50% of an STRF's maximum (if an excitatory HU) or of its minimum (if an inhibitory HU). Each panel in column C only shows a sub-region of the full spectrotemporal range of the STRFs; it is an expansion of an area of interest, whose frequency range and temporal range are shown by the black bars on the edges of the first HU STRF of the neuron. (D) Top: the variance of weighted-output, the input to the output unit (OU), of each HU. The red line marks a variance of 5%, the threshold for distinguishing effective HUs from their “ineffective” counterparts. Bottom: OU nonlinearities for the NRF model for the same 10 cortical neurons. Format as bottom panel in A.

We first qualitatively examined the fitted characteristics of the two models (Fig 3; each of the 10 numbered rows shows an example neuron; neuron 1 was used in Figs 1 and 2B). In our dataset, as is quite commonly the case, the LN model STRFs are rather “punctate”, i.e. the model neuron is driven almost exclusively by stimulus elements clustered narrowly in frequency and recent stimulus history, often an excitatory point with some weak lagging inhibition (Fig 3A, the top panel shows the STRF for each neuron). Moreover, the LN model tends to operate in the near-threshold region of the nonlinear activation function (Fig 3A, lower panel for each neuron), with activations straddling the expansive part of the sigmoidal output function.

The NRF model reveals more complex tuning properties (Fig 3B–3D, for the same 10 example neurons). Each NRF model had 20 HUs, but because the model training incorporated a regularization term that penalizes ineffectual and redundant synaptic weights (see Materials and Methods), HUs could develop substantive synaptic weights only if these were able to “explain” aspects of the firing of the biological neuron that were not already covered by the other HUs in the feedforward network. Any HUs that were redundant would have their input and output weights, and hence their overall contribution to the NRF, shrink to negligibly small values. We found that, of the 20 HUs in each NRF, most turned out to be redundant during the course of model fitting, and each NRF ended up with a relatively small number of “effective” HUs (between 1 and 7), which were the only ones to send strong signals to the output neuron (Fig 3B; for each neuron, each column shows an 'effective' HU with an STRF, top panel, and a nonlinearity, bottom panel). The variance, calculated over the full stimulus set, of an HU's weighted-output (HU output × output weight) provides a measure of the HU's 'effectiveness' (Fig 3D, top panel), with HUs with a variance ≥5% (Fig 3D, red line) of the sum of the variances of all 20 HUs being considered effective. The weighted-output of an effective HU varies greatly as it rises and falls to signal the presence or absence of particular stimulus features. In contrast, the weighted-output variance of an “ineffective” HU is close to zero. Thus, an NRF with three effective HUs (Fig 3B, neuron 1) has three weighted-output variances above the 5% threshold (Fig 3D, top panel, neuron 1).

HUs can be classified into excitatory or inhibitory units according to whether their output weight is positive or negative, after adjusting the model to account for HUs where the input weights are predominantly negative and the output weight negative, which is effectively an excitatory HU, and also adjusting for HUs that show the converse (see Materials and Methods). If the plotted line of an HU's nonlinear activation function is red, it is excitatory, whereas if it is blue, the HU is inhibitory (Fig 3B, bottom panel for each HU). The STRFs of HUs are more diverse in form than the STRFs of the LN model, and together appear to cover a wider range of frequencies and times (Fig 3B, top panel for each HU). For display purposes only, the weights of the inhibitory HU STRFs in Fig 3 have their signs inverted so as to show their influence on the OU (see Materials and Methods, “The displayed STRFs”).

We can examine how the HUs interact if we “zoom in” on a part of the STRF's frequency and temporal range marked by high levels of sensitivity (Fig 3C, 'zoomed' region identified by the black bars along the axes of the STRF for the first HU of each neuron in Fig 3B). Contours delineate the time-frequency regions of high sensitivity for each of the effective HUs, using shades of red for excitatory HUs and shades of blue for inhibitory HUs (Fig 3C). Here, for each excitatory HU, time-frequency regions of high sensitivity were defined as those for which the STRF's weights (as shown in Fig 3C) exceeded half the maximum weight. For the inhibitory effective HUs, high-sensitivity regions were where the STRF weights fell below half the minimum weight. For many neurons, the high-sensitivity regions of the STRFs for different HUs align in close but distinct locations in spectrotemporal space to form apparent structures, suggesting the presence of conjunctive sensitivity to ordered features (see Discussion).

OUs tend to operate “near threshold” (where “threshold” is the lowest possible output value, see Materials and Methods), with activations (Fig 3D, bottom panel per neuron, black histogram) mostly confined to the expansive part of their nonlinear activation function (Fig 3D, bottom panel, red line), just like the LN model. However, the same is not always true for the effective HUs, many of which experience activations (Fig 3B, bottom panels per neuron, black histogram) that sometimes fall in the linear range of their nonlinear activation function (Fig 3B, bottom panels, red or blue line), or even operate over the compressive, upper range of their nonlinear activation function.

### NRF models have between 1 and 7 effective hidden units

**Fig 4:**
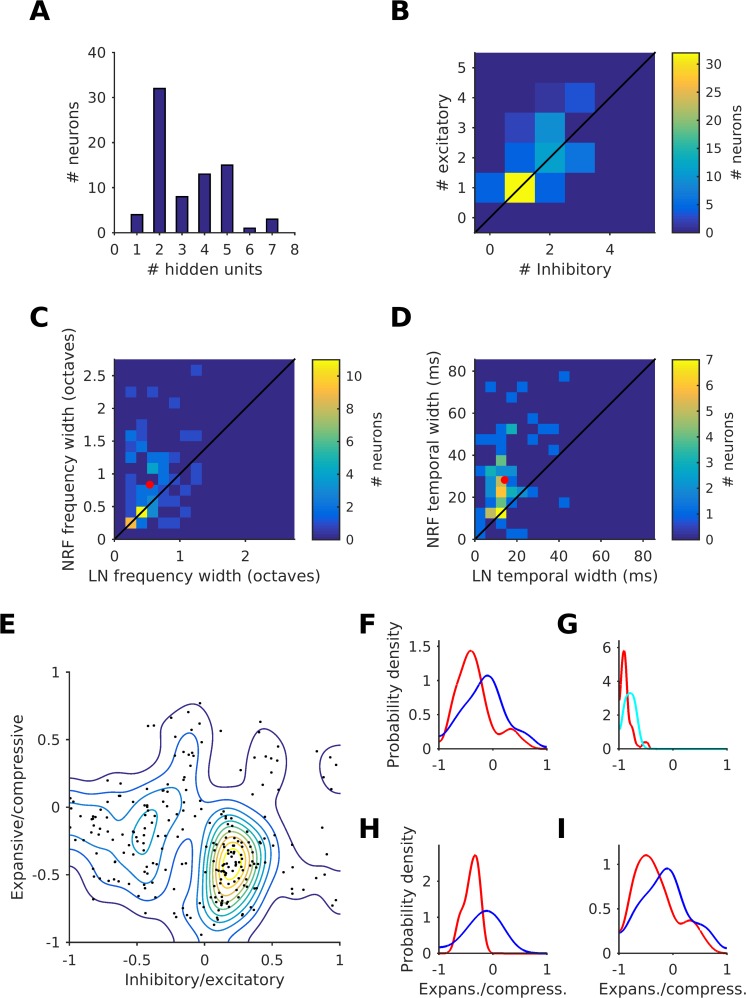
Properties of the feedforward neural network model. (A) Histogram of the number of effective hidden units (HU) of the NRF model fits for each neuron. (B) Distribution of the number of effective excitatory and inhibitory HUs for each neuron. (C) Frequency tuning width of the NRF model versus that of the LN model. The red dot indicates the average frequency tuning width of both models. (D) Temporal tuning width of the power STRF of the NRF model versus that of the LN model. The red dot indicates the average temporal tuning width of both models. (E) A plot of the expansive/compressive (EC) score (which measures how the nonlinear activation function is used) against the excitatory/inhibitory (IE) score for all 246 effective HUs from the 76 neurons. Contour plot shows the density. (F) Distribution of the EC score for excitatory (red) and inhibitory (blue) HUs. (G) Distribution of the EC score for the output unit of the NRF model (red) and for the LN model (cyan). (H) Distribution of the EC score for excitatory (red) and inhibitory (blue) HUs for the bi-feature neurons. (I) Distribution of the EC score for excitatory (red) and inhibitory (blue) HUs for the multi-feature neurons.

For the NRF model, the most common number of effective HUs of a neuron was 2; this was the case for 42% (32/76) of neurons (Fig 4A). Such 'bi-feature' neurons always have one excitatory and one inhibitory HU (Fig 4B). A few 'uni-feature' neurons, with only an excitatory effective HU, made up 5% (4/76) of our sample (Fig 4A). The remaining 53% (42/76) were 'multi-feature' neurons with between 3 and 7 (mode = 5) effective HUs (Fig 4A), which tended to have more excitatory HUs than inhibitory HUs (p = 0.035, n = 76, sign test; Fig 4B).

### NRF models reveal wider integration over time and frequency than LN models

To quantify the time and frequency tuning widths of LN model STRFs, we first calculated the “power STRF” for each neuron by squaring the weights in the STRF (see Materials and Methods). The temporal tuning width was then determined by summing the power STRF over the frequency bands and counting the number of time bins with power ≥25% of the maximum. Multiplying this count by the temporal bin size gave the temporal tuning width at quarter-height. The frequency tuning width at quarter-height was measured analogously by summing the power STRF over time and multiplying the number of bins exceeding a quarter of the maximal power by the width of each frequency channel.

To obtain comparable measurements of overall tuning widths for the NRF models, we calculated power STRFs for each of the NRF model’s HUs, then summed their power STRFs, weighted by the strength of the signals that they contribute to the OU, which was quantified as the variances of their weighted-outputs, as shown in the bar charts in Fig 3D. Quarter-height frequency and temporal tuning widths were then calculated from the weighted sum power STRF in the same way as for the LN model power STRFs.

The quarter-height frequency and the temporal tuning width for the NRF model was, for most neurons, significantly larger than for the LN model (n = 76, p = 1.6×10^−3^ and p = 1.8×10^−12^, respectively, sign-test; Fig 4C and 4D respectively). On average, the frequency tuning width for the NRF model was 0.83 octaves, which is 54% larger than the 0.54 octaves for the LN model. The average temporal tuning width for the NRF model was 28.2 ms, which is 99% larger than the 14.2 ms for the LN model. We also carried out analogous analyses for frequency and temporal tuning widths measured at half-height, which produced similar results, showing significantly larger tuning widths in the NRF model for both frequency and time (27% and 46% larger, respectively, n = 76, p = 0.033 and 1.7×10^−7^, respectively, sign-test).

### Inhibitory and excitatory receptive sub-fields have different nonlinear properties

We next examined whether excitatory or inhibitory HUs differ in the extent to which they operate over the expansive, linear or compressive part of their output nonlinear activation function. For each effective HU in our dataset, we computed an expansive/compressive measure (EC score) and inhibitory/excitatory measure (IE score; see Materials and Methods for details). Both EC and IE scores are bounded between -1 and 1. A unit with a negative EC score operates predominantly in a near-threshold, expansive region of its nonlinearity, while a positive score indicates that it operates in a compressive, saturating region, and a score close to zero indicates operation in a linear region. Negative IE scores mean that a unit is (predominantly) inhibitory and positive scores that it is excitatory. EC scores are plotted against IE scores for all 246 effective HUs from the 76 neurons in Fig 4E. Superimposed on the scatter plot is a contour plot reflecting the density estimate of the scatter in the “EC/IE space”. The density estimation used a kernel with its bandwidth optimized to smooth away statistically spurious peaks [32]. The HUs fell into two broad clusters: the first, dense cluster is excitatory (IE ≥ 0) and expansive (EC < 0), whereas the other, broader cluster is inhibitory (IE < 0) and more linear (EC ≈ 0).

To confirm this observation, we divided the neurons into excitatory (IE ≥ 0) and inhibitory (IE < 0), and separately plotted the distribution of the EC score for each, again using a kernel density estimator with optimally chosen kernel bandwidth (Fig 4F). The EC scores of the 115 inhibitory HUs (blue) were more or less symmetrically distributed around 0, indicating that these HUs mostly operate in a linear region, whereas the great majority of the 131 excitatory HUs have EC scores <0, indicating that they tend to operate in the near threshold, expansive region of their output nonlinear activation function. The difference in the median EC value of the two distributions was significant (p = 6.1***×***10^−6^, rank sum test), confirming the EC/IC space clustering observations (Fig 4E). The distribution of EC scores for the OUs of the NRF model for all 76 neurons (Fig 4G, red), shows that the OUs operate largely near threshold, in the expansive region (EC < 0). This is similar to the case for the EC scores in the LN model (Fig 4G, cyan), where all the neurons also operate in the expansive region (EC < 0).

If we restrict the above analysis to only the HUs of uni-feature and bi-feature neurons (Fig 4H, for density estimation the bandwidths from Fig 4E were used), we observe that the median EC values of the 36 excitatory and 32 inhibitory HUs differ significantly (p = 4.8***×***10^−5^, rank sum test), as is the case for all HUs (Fig 4F). If we restrict the EC distribution analysis to multi-feature neurons alone we again observe the same pattern (p = 2.4***×***10^−3^, rank sum test) as for all HUs (Fig 4I, again the Fig 4E bandwidths were used). However, the 36 excitatory HUs for the uni-feature and bi-feature neurons are significantly (p = 1.7***×***10^−6^, Levene's test) more tightly clustered than the 95 excitatory HUs of multi-feature neurons, indicating that the uni- and bi-feature neurons show less diversity in their use of the nonlinear activation function. The decrease in diversity for the 32 inhibitory HUs of uni- and bi-feature neurons relative to the 83 inhibitory HUs of the multi-feature neurons is also significant (p = 0.032, Levene’s test).

### Potential functional role of nonlinear characteristics

We have seen that NRF models capture more of the response properties of auditory cortical neurons than conventional LN models, and that they achieve this through the interplay of modest numbers of excitatory and inhibitory HUs. In this section, we consider which functional properties of cortical neurons might be captured by the NRF models. We identify two such properties: gain control and multi-feature sensitivity.

#### Gain control

**Fig 5:**
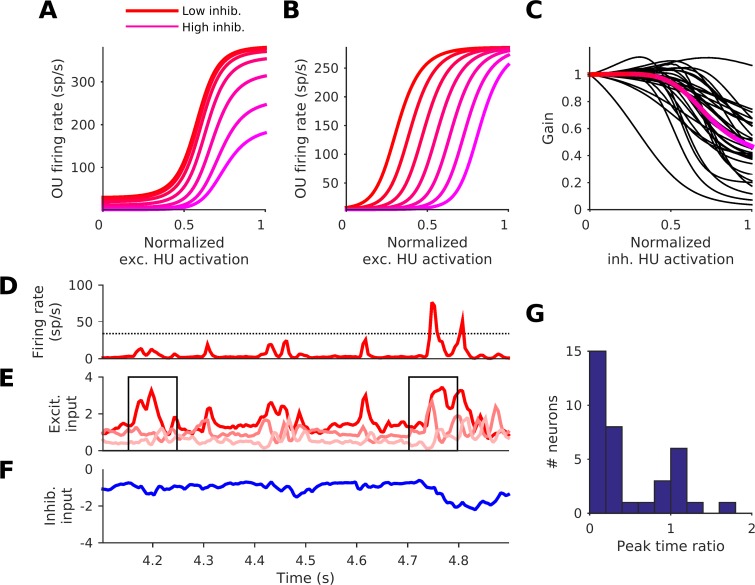
Functional implications of the neural network model fits. (A) The effect of activation of the inhibitory hidden unit (HU) on the relationship between output unit (OU) firing rate and excitatory HU activation, for an example bi-feature neuron's NRF. The steepest slope of each curve is its gain. Activation in all plots is normalized to span 0–1, where 0 is the 1^st^ centile and 1 the 99^th^ centile of the distribution of activations over the stimulus set (Fig 3C). (B) The effect of inhibiting the excitatory HU (instead of the OU) on the same relationship for the same example NRF. (C) The gain as a function of inhibitory HU activation for all 32 bi-feature neurons (black lines). For each neuron the gain is normalized to be 1 when the normalized inhibitory HU activation is 0. Red/magenta line: the mean. Note that the full range of excitatory HU activations, from threshold to saturation of the HU, was examined to find the steepest slopes and hence the gain (i.e. beyond the 1^st^ and 99^th^ centiles). (D) The OU firing rate of an NRF model fit for an example multi-feature neuron (red line). The dotted line is the 2*σ*-threshold. (E) The weighted-output (the input to the OU, HU output × output weight) from the 3 effective excitatory HUs. (F) The weighted-output from the single effective inhibitory HU for this neuron. (G) The distribution of peak time ratio for all multi-feature neurons. The peak time ratio is the number of times the sum of the outputs of all reduced-NRF models exceeded the 2*σ*-threshold, relative to the number of times the output of the full NRF did so. The reduced-NRF models of a neuron each retain just one of the excitatory HUs.

We can use simulations derived from our modeling to show that the interplay of excitation and inhibition seen in the NRF models enables them to exhibit gain control. For an example bi-feature neuron (neuron 2 of Fig 3), we plotted the OU firing rate as a function of the activation of the excitatory HU (Fig 5A). Using different hues from red to magenta, this dependence of OU firing rate on excitatory HU activation is shown for 7 different levels of inhibitory HU activation, which were also chosen to span the range of the inhibitory HU activation. Observe that increasing the inhibitory drive reduces the slope of the relationship between excitatory drive and OU firing rate. In other words, the inhibitory input reduces the gain of the excitatory drive on the OU: the effect of the inhibition is more “divisive” than “subtractive”. This form of gain control is not observed if we feed the inhibition into the excitatory HU instead of the OU; instead a threshold shift, i.e. a “subtractive inhibition”, is seen (Fig 5B). Gain control is also not observed if the HU output functions are linear rather than sigmoidal. We measure the gain as the steepest slope of the OU-firing-rate vs. excitatory-HU-activation curves (Fig 5A). For the bi-feature neurons the gain tends to decrease with increasing inhibitory HU activation (Fig 5C), with the gain for 31/32 neurons being lower for the highest inhibitory HU activation than it is for the lowest inhibitory HU activation.

#### Multi-feature selectivity

We have seen that NRFs can reveal multiple excitatory fields of several HUs arranged closely together, but distinct, in time-frequency space (Fig 3C). This raises the question of what advantage parsing these excitatory regions out over several HUs rather than just combining them in a single STRF, as in an LN model, would bring. To address this, we have plotted the activity of the components of the NRF of one multi-feature neuron (neuron 5 in Fig 3) during 800 ms of test-set auditory stimulation. Plotted are the OU output (Fig 5D) and effective HU weighted-outputs (excitatory HUs, Fig 5E, inhibitory HUs, Fig 5F). Note that the weighted-outputs of the excitatory HUs are often correlated, and that the OU tends to give a substantial response only when the weighted-output of more than one excitatory HU peaks at the same time. For example, at 4.2 s (Fig 5E, left black box), one of the excitatory HUs is highly active, but the other two are not, and the OU gives little response, while at 4.75 s (Fig 5E, right black box) two HUs are active and the OU response is ~6 times higher.

Given that the output and excitatory HUs tend to operate over the expansive, near-threshold range of the nonlinear activation function, one might expect a conjunctive effect to be common, whereby the OU “goes substantially above threshold” only when several features of the excitatory HUs occur together. To examine how single excitatory HUs on their own can drive the OU, we ran the natural sounds through the NRF model with all but one of its excitatory HUs disabled (set below 'threshold'). We did this for each excitatory HU in turn to obtain the response of the OU if it only had that one excitatory HU. Then, to provide a conservative comparison with the original model, we summed the OU response for all of those single excitatory HU reduced-NRF models (for example, a 3 excitatory HU NRF would produce 3 single excitatory HU reduced-NRF models, whose responses were summed). We then determined the number of time bins for which this summed response was above the 2*σ*-threshold (Fig 5D, as in Fig 2B), and called this number the summed-reduced-NRF peak time. We compared this to the number of time bins during which the response of the original model exceeded the 2*σ*-threshold; we called this number the NRF peak time. We did this for all 36 neurons with more than one excitatory HU. Across the 36 neurons, the NRF peak time typically (for 78%, 28/36, of the neurons) exceeded the summed-reduced-NRF peak time (p = 1.2×10^−3^, sign test). Fig 5G shows the distribution over the neurons of the peak time ratio, the neuron's summed-reduced-NRF peak time (the number of times the summed single excitatory HU response was above the 2*σ*-threshold) divided by its NRF peak time (the number of times the 2*σ*-threshold was surpassed by the unmodified model). Here we can see the strength of the conjunctive effect, the summed single excitatory HU response exceeded the 2*σ*-threshold less than half as often as the unmodified model alone (peak time ratio < 0.5) for 67% (24/36) of the multi-feature neurons. This implies that, for many of the multi-feature neurons, spectrotemporal features often interact in a conjunctive, supra-additive manner. Such neurons require the simultaneous presence of multiple particular features to produce a substantial response, and respond very little to just one such feature alone. This need not have been the case, as it could have been that each feature alone could substantially drive the neuron, as is seen for 22% (8/36) of the neurons, which have a peak time ratio ≥1.

## Discussion

The network models we developed here represent a substantial improvement over conventional LN models in that they are able to produce more accurate predictions of the responses of cortical neurons to natural sounds, while remaining sufficiently parsimonious that they it can be quickly fitted using limited data and interpreted in a manner relevant to the known physiology of the auditory pathway. The NRF models are significantly more complex than LN models–in fact, their number of degrees of freedom is greater in proportion to the number of HUs in the network. Nevertheless, compared to the enormous complexity of the lemniscal auditory pathway, in which individual neurons receive potentially thousands of converging inputs, the model complexity remains very modest. Accordingly, and as with all models of cortical processing, we cannot expect the artificial neural network to replicate the biological network in any strict anatomical detail. Instead, given their capacity to predict the responses of auditory cortical neurons to natural sounds, we propose that the NRF models capture important aspects of the general signal processing performed by the neural circuitry driving the recorded neurons, and that this is likely to apply to other areas of the brain too.

### Primary auditory cortical neurons are nonlinearly sensitive to a broad spectrotemporal domain

Our results indicate that auditory cortical neurons likely integrate more widely over time and frequency than linear STRF or LN models would suggest (Fig 4C and 4D). That this integration is highly nonlinear may be the reason why linear STRFs do not effectively measure this broad tuning. Although they can be quite complex [12], LN model cortical STRFs tend to be relatively simple in structure [13]. Given the extensive network that constitutes the central auditory pathway, it seems likely that more sophisticated processing is being carried out than implied by linear STRFs. NRF models may help to shed light on the nature of this processing, as they reveal a diversity, complexity and breadth of spectrotemporal integration well beyond that which can be described by conventional LN models.

One consequence of this finding relates to models of sparse representation of natural sounds [33], a hypothesized method by which the brain may perform unsupervised learning of the statistical structure of the environment. These sparse models result in projective fields (for many parameter settings) that are often broad in frequency and particularly in time. Our results suggest that many neurons with punctate STRFs may be better described by NRF models with broader tuning, which is more consistent with these sparse representational models.

### The neurons are well characterized by 1–7 features that segregate into inhibitory and excitatory features

Although we found that many of our cortical neurons can be characterized as bi-feature (one inhibitory and one excitatory HU), we also found many multi-feature neurons, with 3–7 effective HUs, typically with slightly more excitatory HUs than inhibitory (Fig 4A and 4B). It is interesting to speculate that the bi-feature neurons may mostly be located in granular cortical layers and the multi-feature neurons in the supra/infragranular layers, since the former receive most of the thalamic inputs and are known to show simpler tuning properties than neurons in the supra/infragranular layers [34].

The features (HU STRFs) naturally segregate into those that inhibit the neuron and those that excite it (Fig 4E). The excitatory features tend to operate in the expansive part of the NRF model’s nonlinear activation function, ‘near threshold’, whereas the inhibitory features tend to operate in the more linear part of the nonlinear activation function (Fig 4F). We speculate that the excitatory and inhibitory HUs may reflect the massed effects on the recorded neuron of directly connected excitatory neurons and inhibitory neurons, respectively. Should this be the case, the difference in nonlinear characteristics may reflect the observation that inhibitory neurons tend to have higher evoked and spontaneous firing rates than excitatory neurons [35], thus placing inhibitory inputs further above threshold than excitatory inputs and perhaps providing them with a more linear dependence on input.

### Bi-feature neurons

Many (42%) of the neurons showed NRF fits with just two effective HUs, one excitatory and one inhibitory. The excitatory HU operates near threshold (expansive), and the inhibitory HU is more linear. The OU is also expansive. Under this arrangement, the inhibition acts on the output in a manner that appears to decrease the gain (Fig 5A–5C). A number of possible mechanisms, which might work in isolation or together, have been proposed for gain control, including synaptic depression [36], shunting inhibition [37] and recurrent connectivity [38]. The NRF model illustrates another possible mechanism—feedforward expansive excitation and feedforward linear inhibition acting together on a neuron with an expansive nonlinearity. In vivo patching approaches may provide a method to explore this possibility, since it may be possible to measure the inhibition and excitation separately, as well as assess the output nonlinearity. The above discussion prompts two modifications to the model to be examined in future work. The first is to include some explicit gain control mechanism, for example, HUs with a divisive effect as a functional model of shunting inhibition. The second is to add an additional layer, which will allow for HUs to depend more directly on nonlinear measures like the standard deviation of the stimulus and perhaps capture the use of gain control to normalize for contrast, as has been observed for auditory neurons with artificial stimuli [19,39–41].

### Multi-feature neurons

The multi-feature neurons are quite a diverse group, and substantially larger population samples would therefore be needed to look for trends in their properties and investigate whether they form identifiable groups that may serve distinct purposes. However, we can make a number of observations. Although the HUs of multi-feature neurons show more diverse nonlinearity characteristics than bi-feature neurons, they still tend towards having expansive-range excitatory HUs and linear-range inhibitory HUs. The set of STRFs of multi-feature neurons can be quite complex and varied (Fig 3B), and can show distinctly structured relationships between these STRFs (e.g. neurons 7 and 8, Fig 3C). Often the spectrotemporal regions of high sensitivity (half-height tuning area) of HU STRFs do not substantially overlap (e.g. neurons 1, 4 and 9, Fig 3C). However, some overlap of high sensitivity regions in the STRFs can occur, between excitatory HUs (e.g. neurons 5–8, Fig 3C), between inhibitory HUs (neuron 7, Fig 3C), and between excitatory and inhibitory HUs (neurons 6, 7, and 10, Fig 3C).

Given that the model fitting process penalizes redundant STRF weights, the presence of spectrotemporal overlap in STRFs of different HUs may indicate that the NRF is using multiple HUs to alter the nonlinearity of the input-output mapping in order to achieve a better fit to the true output nonlinearity of the biological neuron. However, for the most part, the high sensitivity regions of HU STRFs are non-overlapping, suggesting that additional factors drive the diversity of multi-feature neuron STRFs. In a number of cases, excitatory fields of different HUs align consecutively along the time axis, sometimes with some overlap (e. g. neurons 4, 6, 7 and 10, Fig 3C). This may to some extent capture the relationship between sound intensity and response latency found in both the auditory nerve and the cortex [42], as in some cases the shorter latency HU also has a higher ‘threshold’ (i.e. has a lower EC value, e.g. neuron 4, Fig 3C). However, this is unlikely to be the whole story, because in other cases different excitatory HUs exhibit distinct well-separated regions of temporal tuning (e.g. neuron 10, Fig 3C). In addition, STRF excitatory fields may also align over the frequency axis (e.g. neurons 1, 5 and 8, Fig 3C) or align diagonally over time and frequency (e.g. neurons 6 and 9, Fig 3C).

For many multi-feature neurons (although far from all), the NRF requires that multiple excitatory HUs are activated simultaneously to produce a substantial response (Fig 4D–4G). That the NRF model can capture such supra-additive sensitivity to particular conjunctions of multiple spectrotemporal features, while the LN model cannot, may explain why the NRF model is better able to predict the peak amplitudes of the responses of cortical neurons. This conjunctive feature selectivity allows for increased selectivity for particular complex spectrotemporal patterns consisting of a number of more basic features, a characteristic with an obvious potential role in sound recognition.

### Related work

A number of methods have been used previously to examine the spectrotemporal sensitivity of auditory cortical neurons. Previous studies have attempted to extend the application of the LN model to auditory cortical data, mostly using maximum-likelihood methods. Indeed, several studies have used approaches that have fundamental similarities to the one we explore here, in that they combine or cascade several linear filters in a nonlinear manner. One such body of work that improved predictions over the LN model is based on finding the maximally-informative dimensions (MID) [20,21,34,43–46] that drove the response of auditory cortical neurons. This method involves finding usually one or two maximally informative linear features that interact through a flexible 1D or 2D nonlinearity, and is equivalent to fitting a form of LN model under assumptions of a Poisson model of spiking variability [46–48]. When this method was applied to neurons in primary auditory cortex it was found that the neurons’ response properties are typically better described using two features rather than one [20,34], in contrast to midbrain neurons which are well fitted using a single feature [43]. That result thus seems consistent with ours, in that we found NRFs fitted to cortical responses most commonly evolved to have two effective HUs (or input features). Another approach, that has been found to improve predictions of auditory cortical responses, is to apply a multi-linear model over the dimensions of frequency, sound level, and time lag, and for the extended multi-linear model also over dimensions involved in multiplicative contextual effects [21]. However, the above studies in auditory cortex [20,34,43] did not use natural stimuli, and hence might not have been in the right stimulus space to observe some complexities, as STRFs measured with natural stimuli can be quite different than when measured with artificial stimuli [49]. An advantage of the NRF model is that its architecture is entirely that of traditional feedforward models of sensory pathways in which activations of lower level features simply converge onto model neurons with sigmoidal input-firing rate functions. NRFs can therefore be interpreted in a context that is perhaps simpler and more familiar than that of, for example, maximally informative dimension models [20,44].

Other developments on the standard LN model have included model components that can be interpreted as intraneuronal rather than network properties, such as including a post-spike filter [22] or synaptic depression [23], and have also been shown to improve predictions. Pillow and colleagues [50,51] applied a generalized linear model (GLM) to the problem of receptive field modelling. Their approach is similar to the basic LN model in that it involves a linear function of stimulus history combined with an output nonlinearity. However, unlike in LN models, the response of their GLM also depends on the spike history (using a post-spike filter). This post-spike filter may reflect intrinsic refractory characteristics of neurons, but could also represent network filter effects. A GLM model has been applied to avian forebrain neurons [22], where it has been shown to significantly improve predictions of neural responses over a linear model, but not over an LN model.

Although they haven’t yet been applied to auditory cortical responses, it is worth mentioning two extensions to GLMs. First, GLMs can be extended so that model responses depend on the history of many recorded neurons [50], representing interconnections between recorded neurons. While this approach is thus also aimed at modeling network properties, it is quite different from our NRF model, where we infer the characteristics of hidden units. Second, the extension of the GLM approach investigated by Park and colleagues [52] included sensitivity to more than one stimulus feature. Thus, like our NRF or the multi-feature MID approach, this “generalized quadratic model” (GQM) has an input stage comprising several filters which are nonlinearly combined, in this case using a quadratic function. One might argue that our choice for the HUs of a sigmoidal nonlinearity following a linear filter stage, and the same form for the OU, is perhaps more similar to what occurs in the brain, where dendritic currents might be thought of as combining linearly according to Kirchhoff’s laws as they converge on neurons that often have sigmoidal current-firing rate functions. However, we do not wish to overstate either the physiological realism of our model (which is very rudimentary compared to the known complexity of real neurons) or the conceptual difference with GQMs or multi-feature MIDs. A summation of sigmoidal unit outputs may perhaps be better motivated physiologically than a quadratic function, but given the diversity of nonlinearity in the brain this is a debatable point.

Another extension to GLMs, a generalized nonlinear model (GNM), does, however, employ input units with monotonically-increasing nonlinearities, and unlike multi-neuron GLMs or GQMs, GNMs have been applied to auditory neurons by Schinkel-Bielefeld and colleagues [24]. Their GNM comprises a very simple feedforward network based on the weighted sum of an excitatory and an inhibitory unit, along with a post-spike filter. The architecture of that model is thus not dissimilar from our NRFs, except that the number of HUs is fixed at two, and their inhibitory and excitatory influences are fixed in advance. It has been applied to mammalian (ferret) cortical neural responses, uncovering non-monotonic sound intensity tuning and onset/offset selectivity.

For neurons in the avian auditory forebrain, although not for mammalian auditory cortex, GNMs have also been extended by McFarland and colleagues to include the sum of more than two input units with monotonically-increasing nonlinearities [53]. Of the previously described models, this cascaded LN-LN ‘Nonlinear Input Model (NIM)’ model bears perhaps the greatest similarity with our NRF model. Just like our NRF, it comprises a collection of nonlinear units feeding into a nonlinear unit. The main differences between their model and ours thus pertain not to model architecture, but to the methods of fitting the models and the extent to which the models have been characterized. The NIM has been applied to a single zebra finch auditory forebrain neuron, separating out its excitatory and inhibitory receptive fields in a manner similar to what we observe in the bi-feature neurons described above.

One advantage of the NRF over the NIM is that the fitting algorithm automatically determines the number of features that parsimoniously explain each neuron's response, obviating the need to laboriously compare the cross-validated model performance for each possible number of hidden units. Another difference is that the NRF is simpler while still maintaining the capacity to capture complex nonlinear network properties of neural responses; for example, the NIM [53] had potentially large numbers of hyperparameters (four for each hidden unit or “feature”) that were manually turned, something that would be very difficult to do if the model needed to be fitted to datasets comprising large numbers of neurons. In contrast, the NRF has only one hyperparameter for the entire network, which can easily be tuned in an automated parameter search with cross-validation. Consequently, we have been able to use the NRF to characterize a sizeable population of recorded neurons, but so far no systematic examination of the capacity of the NIM to explain the responses of many neurons has been performed.

Another recent avian forebrain study [54] used a maximum noise entropy (MNE) approach to uncover multiple receptive fields sensitive to second-order aspects of the stimulus. Unlike the above two GNM [24,53] approaches, this model does not have hidden units with sigmoidal nonlinearities, but finds multiple quadratic features. The MNE predicted neural responses better than a linear model, although still poorly, with an average *CC*_*raw*_ of 0.24, and it was not determined whether it could out-predict an LN model. Note, however, that the *CC*_*raw*_ values reported in that study do not distinguish stimulus-driven response variability from neural “noise”. Consequently, it is unclear whether the relatively modest *CC*_*raw*_ values reported there might reflect shortcomings of the model or whether they are a consequence of differences in the species, brain regions and stimuli under study. Finally, perhaps the most relevant study in the avian forebrain used a time delay feedforward neural network to predict responses of zebra finch nucleus ovoidalis neurons to birdsong [55]. These authors reported that the network predicted neural responses better than a linear model, but performed no quantitative comparisons to support this.

Advances on the LN model have also been applied in other brain regions. Various advances on the LN model have also been made in studies of primary visual cortex, and of particular relevance are the few cases where neural networks have been used to predict neural responses. Visual cortical responses to certain artificial stimuli (randomly varying bar patterns and related stimuli) have been fitted using a single hidden layer neural network, resulting in improvements in prediction over linear models for complex but not simple cells in one study [56] and over LN-like models in another study [57]. However, the challenge we tackle here is to predict the responses to natural stimuli. In this respect we are aware of only one similar study by Prenger and colleagues [58] which used a single hidden layer neural network to predict responses to series of still images of natural scenes. The network model in this study gave better predictions than an LN model with a simple rectifying nonlinearity. However, the improvements had limited consistency, predicting significantly better in only 16/34 neurons, and it did worse than an LN model applied to the power spectra of the images. Additionally, the *CC*_*raw*_ of the model predictions with the neural data were somewhat small (0.24). This appears to contrast with the seemingly better performance we obtained with our NRF model.

These apparent differences in model performance may, however, not all be attributable to differences in model design or fitting. In addition to the fact we already noted that low *CC*_*raw*_ values might be diagnostic of very noisy neurons rather than shortcomings of the model, we also need to be cognizant of the differences in the types of data that are being modeled: we applied our model responses of auditory cortical neurons to natural auditory sound recordings, whereas Prenger and colleagues [58] applied theirs to visual cortical neuron responses to random sequences of photographs of natural scenes. Furthermore, the neural responses to our stimuli were averaged over several repeats, whereas the above study did not use repeated stimuli, which may limit how predictable their neural responses may be. However, there are also notable structural differences between their model and ours. For example, the activation function on the OU in the Prenger et al. study [58] was linear (as with [56] but not [57]), whereas the OU of our NRF has a nonlinear activation function, which enables our NRF to model observed neuronal thresholds explicitly. Furthermore, we used a notably powerful optimization algorithm, the sum-of-function optimizer [26], which has been shown to find substantially lower values of neural network cost function than the forms of gradient descent used in the above neural network studies. Finally, the *L*_1_-norm regularization that we used has the advantage of finding a parsimonious network quickly and simply, as compared with the more laborious and often more complex methods of the above three studies: *L*_2_-norm-based regularization methods and hidden unit pruning [58], early stopping and post-fit pruning [56] or no regularization and comparing different numbers of hidden units [57].

### Predictive capacity and possible model improvements

The predictions of the NRF models correlate with the observed neural responses with a *CC*_*norm*_ of 0.73 on average. Asari and Zador [31] estimated an upper limit on the performance that any model of A1 neurons might be able to achieve in predicting responses from a given duration of stimulus history. Our models predict responses from the last 100 ms of stimulus history, for which Asari and Zador [31] give an upper performance limit of 0.5–0.55 “signal power explained” (SPE). For SPE values in this range, SPE is approximately equal to the square of *CC_norm_* [59], so that an upper limit SPE of 0.5–0.55 corresponds to an upper limit *CC_norm_* of 0.71–0.74. This suggests that the NRF may possibly be capturing the majority of the neural response that is dependent on the stimulus, given the duration of stimulus history provided (100 ms).

The performance upper bound reaches its maximal plateau when about 3 s of stimulus history are provided [31]. This suggests that the most important way of advancing neural network models of auditory cortex might be to include a substantially longer stimulus history in the analysis. However, simply extending the number of time bins in the current model some 30 fold further into the past would likely lead to far too many free parameters. A better option might be to extend the approach presented here in the direction of convolutional or recurrent neural networks. Artificial recurrent neural networks have been applied successfully to sound recognition problems [60], and it is well known that feedback projections are common features of the auditory pathway. Developing recurrent versions of the NRFs introduced here is therefore likely to be important, particularly if we hope to develop successful models of higher order auditory cortical neurons.

### Conclusions

In summary, we have shown that fitting feedforward network models (with regularization of the weights to be sparse) to single neuron activity in primary cortical areas (A1/AAF) allows for better predictions of their responses to natural sounds, and has the potential to unmask some of the nonlinear signal processing strategies used by the auditory brain. This approach reveals more of the underlying richness and nonlinearity of cortical processing in an easily interpretable form. Neural responses to natural sounds in A1/AAF appear to be dependent on multiple features in the stimulus space that often interact in structured nonlinear ways, and depend upon a substantially larger spectrotemporal domain than is suggested by linear models with a simple output nonlinearity.

## Materials and Methods

### Electrophysiological recording

To assess the capacity of NRFs to account for cortical sensory responses, we fitted models to neural responses to clips of natural sounds. Single-unit responses were recorded with multi-channel electrodes in the ferret primary auditory cortex (A1) and the anterior auditory field (AAF), which are both considered to be primary cortical areas [61]. All animal procedures were performed under license from the United Kingdom Home Office and were approved by the local ethical review committee. For full details of the recording procedures see [62]. In brief, electrophysiological recordings were made from 6 adult pigmented ferrets under ketamine (5 mg/kg/h) and medetomidine (0.022 mg/kg/h) anesthesia. Bilateral extracellular recordings were made in A1/AAF using either 16 or 32 channel silicon probe electrodes (Neuronexus Technologies). Because these primary cortical fields share a common tonotopic gradient [61,63], we did not attempt to assign our sample of 76 units to one or other of these regions.

### Stimuli

In this study we modeled the responses of neurons to 20 clips of natural sound recordings. Each clip was 5 s long, and presented at a sampling rate of 48,828.125 Hz, using earphones as described by [19]. The clips were presented in random order, with a ~1 s silent interval between clips, and were repeated 20 times. The natural sound recordings included animal sounds (e.g. ferret vocalization and birdsong), environmental sounds (e.g. water and wind), and speech. The RMS intensity of clips ranged from 75 to 82 dB SPL. Data recorded during the first 250 ms after the onset of each stimulus were discarded, leaving an effective set of neural responses to 20 repeats of 20 sounds of 4.75 s duration each.

### Preprocessing of neural data and stimuli

NRF and LN models were fitted to the relationship between the neural data and the sound stimuli, after appropriate preprocessing as described below.

#### Neural data

Recorded spikes were sorted offline using Spikemonger, in-house software built around Klustakwik [64], to isolate single units. For each neuron, for each clip, peri-stimulus time histograms (PSTHs) were constructed, counting spikes in 5 ms bins, averaging over all 20 repeats, and subsequently smoothing with a 21 ms wide Hanning window [29] to estimate the spike count PSTH *y*_*n*_(*t*_*n*_) for each neuron at time *t*_*n*_, where *t*_*n*_ is the time since the start of clip *n* (*n* goes from 1 to *N* = 20, *t*_*n*_ from 1 to *T*_*n*_ = 949). For fitting the NRF model the spike counts were also linearly rescaled to span the standard network nonlinear activation function (see below), so spike count 0 mapped to -σ_1_ and spike count 1 to +σ_1_, where σ_1_ = 1.7159. For model comparison, all spike counts were rescaled back, and for display all spike counts were rescaled to spike rates. To identify those neurons that were driven by the stimuli, we calculated a “noise ratio” (NR) statistic for each neuron [19,65] and excluded from further analysis any neurons with a NR>40.

#### Cochleagram

To transform the sound stimuli into a simple approximation of the activity pattern received by the auditory pathway, we processed the sound waveforms to calculate log-scaled spectrograms ('cochleagrams'). For each sound, the power spectrogram was taken using 10 ms Hamming windows, overlapping by 5 ms. The power across neighboring Fourier frequency components was then aggregated using overlapping triangular windows comprising 34 frequency channels with center frequencies ranging from 500 Hz to 22,627 Hz (⅙ octave spacing). Next, the log was taken of the power in each time-frequency bin, and finally any values below a low threshold were set to that threshold. These calculations were performed using code adapted from melbank.m (http://www.ee.ic.ac.uk/hp/staff/dmb/voicebox/voicebox.html). Both the LN and the NRF models were trained to predict the firing rate *y*_*n*_(*t*_*n*_) at time *t*_*n*_ from a snippet of the cochleagram extending 100 ms (20 time bins) back in time from *t*_*n*_. The input to the models at time *t*_*n*_ is thus a 34×20 matrix (*F =* 34 frequency channels by *H =* 20 stimulus history time bins) of log sound power values preceding time *t*_*n*_. We denote this as *x*_*nfτ*_(*t*_*n*_), where *n* is the index of the presented clip, *f* indexes the frequency bands, and *τ* indexes time history bins preceding time *t*_*n*_. For fitting the NRF model, *x*_*nfτ*_(*t*_*n*_) was also normalized so the whole dataset had zero mean (<*x*_*nfτ*_(*t*_*n*_)>_*nfτ*_ *= 0)* and unit variance. To simplify notation we define *t* as all the times *t*_*n*_ of all the sound clips *n*, where *t* goes from 1 to *T*_*n*_ × *N*. This gives *y*(*t*) and *x*_*fτ*_(*t*).

### LN model

#### Linear stage

The LN model (Fig 1A) consists of two stages: a linear STRF followed by a sigmoidal output nonlinearity. The linear part of the model is:

$$\hat{a}\left(t\right)=\sum_{f,\tau }w_{f\tau }x_{f\tau }\left(t\right)+b$$

where $\hat{a}\left(t\right)$ is the model neuron's “activation”, and *w*_*fτ*_ is the synaptic weight for frequency band *f* and history bin *τ* (all the weights compose the STRF). The bias *b* represents the neuron's background activity level. *w*_*fτ*_ and *b* are the free parameters of the model, and were estimated by regressing *y*(*t*) against *xfτ*(*t*) using 'glmnet' [66]. Thus $\hat{a}\left(t\right)$ can be seen as the best linear prediction from *x*_*fτ*_(*t*) of *y*(*t*). To avoid overfitting and to find a parsimonious model, the regression was regularized by penalizing the *L*_1_-norm of *w*_*fτ*_ (LASSO regression). The strength of the regularization was controlled with a hyperparameter *λ*. The optimum value of *λ* was found using k-fold cross-validation for a set of log-spaced values and for each neuron, and the *λ* that gave the best prediction was chosen (see *Training*, *validation*, *and testing of models* below). The resulting $\hat{a}\left(t\right)$ serves as the input to the nonlinear stage for our LN-model, and as the linear prediction (output) of the purely linear L-model used for the model comparisons which are described in Results.

#### Nonlinear stage

The second stage involved fitting a logistic sigmoid nonlinear activation function,

$$\hat{y}(t)=\frac{\rho_{1}}{1+\text{exp}\left(-\left(\hat{a}(t)-\rho_{3}\right)/\rho_{2}\right)}+\rho_{4}$$

which mapped the linear activation $\hat{a}\left(t\right)$ to the predicted PSTH $\hat{y}\left(t\right)$ so as to minimize the error between the predicted PSTH and the observed PSTH *y*(*t*). Recent work [67] indicates that choosing different nonlinear output functions from a wide range of plausible candidates has only modest effects on the ability of LN models to capture neural response properties. We therefore did not attempt to systematically explore different types of output nonlinearity or to make the choice of nonlinearity as physiological as possible, but rather focused on an output nonlinearity that is simple, well characterized and widely used in the artificial network literature. The four parameters ρ_i_ of the function were fitted by minimizing the squared error

$$E=\sum_{t}{\left(\hat{y}\left(t\right)-y\left(t\right)\right)}^{2}$$

using a quasi-Newton iterative numerical method (http://www.cs.ubc.ca/~schmidtm/Software/minFunc.html).

### NRF model

#### Model description

NRFs (Fig 1B) model cortical responses using a rate based feedforward artificial neural network (multilayer perceptron) with one hidden layer of *J* = 20 hidden units (HU) converging onto a single output unit (OU). Each unit in the network operates in a fashion similar to an LN model—each unit integrates inputs through a set of linear weights, and this linear activation is passed through a nonlinear activation function to compute its output. The activation of the *j*-th HU *a*_*j*_(*t*) is,

$$a_{j}\left(t\right)=\sum_{f,\tau }w_{jf\tau }x_{f\tau }\left(t\right)+b_{j}$$

where *w*_*jfτ*_ is the weight from frequency band *f* and time delay *τ* to HU *j*, and *b*_*j*_ is the bias on the HU. The output of the HU is *z*_*j*_(*t*), given by,

$$z_{j}\left(t\right)=g\left(a_{j}\left(t\right)\right)$$

where g(*ζ*) is a nonlinear function. The OU then provides the prediction $\hat{y}\left(t\right)$, of the firing rate *y*(*t*), as a weighted sum of the HU outputs, also passed through the nonlinear activation function. The activation *a*_*o*_(*t*) of the OU is,

$$a_{o}\left(t\right)=\sum_{j}w_{j}z_{j}\left(t\right)+b_{o}$$

where *w*_*j*_ is the weight from HU *j* to the OU, and *b*_*o*_ is the bias on the OU. The output $\hat{y}\left(t\right)$ of the OU is;

$$\hat{y}\left(t\right)=g\left(a_{o}\left(t\right)\right)$$

which is the model’s prediction of the rescaled firing rate (see *Preprocessing of neural data and stimuli*). For both HUs and OUs, the nonlinear activation function g(*ζ*) was a hyperbolic tangent function:

$$g\left(\zeta \right)=\rho_{1}\text{tanh}\left(\zeta /\rho_{2}\right)$$

In the LN model, the parameters σ_i_ of the nonlinear activation function were optimized for each neuron, but in the NRF model these parameters were fixed to *ρ*_1_ = 1/tanh(2/3) ≈ 1.7159 and *ρ*_2_ = 3/2, which ensures that g(±*1*) = ±1. Using this particular form of nonlinear activation function [68] facilitates efficient learning with error backpropagation by maintaining statistical properties of the input distribution. Furthermore, for a given network with tanh activation functions, there is an equivalent network with logistic activation functions (for which units have non-negative outputs), which can be found with a simple linear rescaling of the weights and biases (see page 109 of [69]). This rescaling does not affect the structure of the STRFs. We use this equivalent network for display in the Results (for details see *The adjusted network* below). Note that the nonlinearities g(*ζ*) employed by the NRF and the LN models are equivalent except for a scaling and shifting.

#### Learning

The free parameters of the NRF, *w*_*jfτ*+_, *b*_*j*_, *w*_*j*_ and *b*_*o*_, were optimized by minimizing the following objective function:

$$E=\frac{1}{2}\sum_{t}{\left(\hat{y}\left(t\right)-y\left(t\right)\right)}^{2}+\lambda \left(\sum_{j,f,\tau }^{}\left|w_{jf\tau }\right|+\sum_{j}^{}\left|w_{j}\right|\right)$$

This objective function is the sum of two terms: The first term quantifies total square error between the observed PSTH *y*(*t*) and the PSTH $\hat{y}\left(t\right)$ predicted by the model. The second term, proportional to the sum of the absolute values of all the weights in the network (the *L*_1_-norm of the weight vectors), serves to regularize the weights. That is, it puts a “cost” on non-zero synaptic weights and will tend to drive most weights to close to zero, except for a few, and thereby encourages parsimonious models and prevents overfitting. The regularization was therefore similar to the LASSO regression used to fit the LN model, which also incorporates an *L*_1_-norm regularization term.

For both the NRF and the LN models, the constant *λ* is the hyperparameter that determines the strength of the regularization. Its optimum value was determined using k-fold cross-validation (k = 10) over a log-spaced range, and for each model and neuron the value of *λ* that gave the best prediction for each neuron was chosen (see *Training*, *validation*, *and testing of models*). The NRF was initialized with the weights and biases independently drawn from a uniform distribution between $\pm 1/\sqrt{M}$ where *M* is the number of incoming connection weights and biases to a given unit of the network. The objective function of the NRF model was minimized using the Sum-of-Functions Optimizer, a recently developed algorithm which combines a Newton method with batch stochastic gradient descent, and which is substantially faster and finds lower minima than other optimization algorithms for multilayer feedforward networks [26]. The optimizer was run for 40 iterations, but usually settled within 20. On a desktop PC (Intel Xeon 8-core 3.1GHz CPU) it took on the order of hours to fit all 76 neurons, including the 10-fold cross validation.

### Training, validation, and testing of the models

For both the LN models and the neural networks, the model parameters (weights *w* and biases *b*, and for the LN models also the parameters of the nonlinear activation function *ρ*_*i*_) were found through the model fitting steps just described, but the models can only perform effectively if the model hyperparameters (regularization strength *λ* and, for the NRFs, also the number of HUs *J*) are appropriately chosen. We therefore conducted a parameter search which systematically explored the behavior of the models for a range of hyperparameters in a cross-validation test. To this end, the entire data set of 95 s duration (20 natural sound clips of 4.75 s duration each) was first split into a cross-validation set (80%) and a test set (20%). The test set was the last 20% (0.95 s) of each of the 20 sounds. The test set was put aside. The cross-validation set was then used to determine the hyperparameters by using k-fold cross-validation (k = 10). The cross-validation set was split into a training set (90% of the cross-validation set, that is the first 3.8s of 18 of the sounds) and a validation set (the remaining 10% of the cross-validation set, that is first 3.8s of 2 of the sounds).

The following steps were performed for each neuron and for each model. For a given *λ*, the model was first fitted on the training set, then the fitted model was used to predict the PSTH of the reserved validation set, and the prediction performance quantified by the normalized correlation coefficient (see *Performance measures*). This process was repeated *k = 10* times, each time using a different non-overlapping 10% of the data as a validation set. The above process was performed for a log spaced set of *λ* values. Then the *λ* was chosen that maximized the mean prediction performance of the 10 validation sets. Optimum *λ* differed across neurons (for both LN and NRF models), and over the two models, and was thus set separately for each model and neuron. A similar process was also performed over *J*, the number of HUs, for a number of reasonable *λ* values. However, as NRF prediction performance varied little as a function of *J*, this was simply set to 20 for all neurons.

Then for each neuron, both models were re-fitted to the full cross-validation set, using the optimum *λ* values, and each model was used to predict the PSTH of the test set. The prediction performance of two fitted models was compared using the performance measures described below. These model fits are the ones used throughout the results section.

To verify that the model fits (at the best *λ* for each neuron) were consistent across the ten different cross-validation fits, we quantitatively compared the STRF of the effective HUs obtained for each validation set. For a given neuron, the effective HU from a given fit that was most correlated with the effective HU from a different fit was found on average to share a correlation coefficient of 0.82, while the second most correlated pair of HUs across fits shared a correlation coefficient of 0.69. These high correlation coefficients are indicative of a high degree of consistency. We verified that, in the absence of repeatable fits, one would expect these correlation coefficients to be close to zero by randomly permuting the weights within every effective HU STRF matrix. This randomization caused the correlation coefficients to drop to 0.06 and 0.02 respectively.

### Performance measures

Model performance was quantified using three different performance measures: the normalized correlation coefficient *CC*_*norm*_, the mean squared error *MSE*, and the peak activity mean square error *pMSE*. While the *MSE* is a well known quantifier of “goodness of fit”, the other two require further explanation.

#### Normalized correlation coefficient

*CC*_*norm*_ quantifies model performance relative to a theoretically achievable maximum and independently of physiological noise. We use it as our standard performance measure in this paper, as it has a number of desirable properties [59], including the fact that it discounts the intrinsic noise of neural responses and quantifies the proportion of the stimulus driven response variability that is captured by the model. If the (Pearson's) correlation coefficient *CC*_*raw*_ between observed and predicted responses is low, then this could either indicate that the model is poor, or that the firing of the neuron under study is poorly stimulus driven and thus fundamentally quite unpredictable by a model that relies on stimulus history as the only explanatory variable. *CC*_*norm*_ does not have that shortcoming, and thus provides a more objective measure of model performance. We calculated the *CC*_*norm*_ [29,30] as the ratio of the *CC*_*raw*_ between the model’s predictions $\hat{y}\left(t\right)$ and the real PSTH *y*(*t*), over the maximum correlation coefficient *CC*_*max*_ that is achievable by a perfect model, given the inherent variability of a particular set of neural responses:

$$CC_{norm}=\frac{CC_{raw}}{CC_{max}}$$

*CC*_*max*_ is defined as the correlation coefficient between the PSTH of the recorded dataset constructed from the *R* repeats of the stimulus (here: *R = 20*) and the PSTH for an infinite number of repeats. *CC*_*max*_ cannot be measured directly, but, one can compute good estimates [29,30] of *CC*_*max*_ using the formula:

$$CC_{max}=\sqrt{\frac{2}{1+\frac{1}{CC_{half}}}}$$

Here *CC*_*half*_ is the correlation coefficient of the mean PSTH of *R/2* repeats with the mean PSTH of the remaining *R*/2 repeats. *CC*_*half*_ depends on the particular split of the *R* observations, and in order to minimize error the splitting is repeated many times and the values of *CC*_*half*_ are averaged. We took the average *CC*_*half*_ over a randomly chosen 126 combinations.

#### Peak activity mean square error

The *pMSE* is the *MSE* between the predicted and observed PSTH for those parts of the signal where the observed firing rate is above the “2*σ*-threshold”, defined as two standard deviations above the mean firing rate to the sound. That is:

$$pMSE=\frac{1}{\sum_{t}^{}p\left(t\right)}\sum_{t}p\left(t\right){\left(\hat{y}\left(t\right)-y\left(t\right)\right)}^{2}$$

where *p*(*t*) is a binary window function consisting of all the binary window functions *p*_*n*_(*t*_*n*_) for each clip *n*. The binary window functions isolate the peaks of the signal:

$$p_{n}\left(t_{n}\right)=\left\{\begin{array}{c} 1\;\text{if}\;y_{n}\left(t_{n}\right)\geq \mu_{n}+2\sigma_{n}\\ 0\;\text{if}\;y_{n}\left(t_{n}\right)<\mu_{n}+2\sigma_{n} \end{array}\right\}$$

where *μ*_*n*_ is the average firing rate and *σ*_*n*_ its standard deviation for sound clip *n*. See *Preprocessing of neural data and stimuli* for how *t* relates to *t*_*n*_, put briefly, we define *t* as all the times *t*_*n*_ of all the sound clips *n*.

### Quantifying model properties

#### The adjusted network

While a biological neuron can only produce positive firing rate outputs and its 'synaptic output weights' are either only excitatory or only inhibitory, the nonlinear activation function of the NRF model allows both positive and negative outputs and is symmetric around zero. Likewise, with the NRF the same weight can be either positive or negative. While these aspects of the NRF model thus lack biological realism, they do offer distinct practical advantages. First, there is a considerable literature on how to train this type of multilayer perceptron model efficiently [28,68]. Second, it gives a network the freedom to discover during training how many excitatory or inhibitory neurons it requires, obviating the need to stipulate a fixed set of excitatory HUs and a fixed set of inhibitory HUs from the outset. However, the fact that the output and weights of a NRF unit can span both positive and negative values makes the distinction between excitatory and inhibitory neurons less categorical and somewhat ambiguous. Nevertheless, this can be resolved because an equivalent 'adjusted' network of excitatory and inhibitory units with logistic activation functions (and hence non-negative outputs) can be found, thus overcoming this problem [69]. Hence we can take advantage of the ease of training tanh networks while preserving the interpretability of logistic networks. This 'adjusted network' was used for all results.

In making the adjusted network, we first consider whether a HU is excitatory or inhibitory. The NRF unit output nonlinearities preserve the sign of the unit activation, so whether the *j*-th HU of an NRF has an overall inhibitory or excitatory effect on the OU will not only depend on the sign of the synaptic weight *w*_*j*_ that connects these two units, but also on the sign of the “expected activation” of the HU, which in turn depends on whether the HU’s STRF is composed mostly of negative or positive weights. To give an extreme example, a HU with all negative STRF weights and a negative *w*_*j*_ would have a positive, excitatory influence on the output neuron. Whether a HU should be considered excitatory or inhibitory thus depends on the product of *w*_*j*_ and the sum of the weights of its STRF $\sum_{f,\tau }w_{jf\tau }$. If both are positive or both negative, the HU is excitatory, otherwise it is inhibitory. This is potentially confusing when readers are used to the idea that, whether a HU is inhibitory or not, can simply be determined from the sign of its output synaptic weight. Note, however, that the equation governing the NRF model unit’s nonlinearity is symmetric and odd, so that for HU *j*, multiplying *w*_*j*,_ *b*_*j*,_ and *w*_*jfτ*_ by -1 leaves the influence of that HU on the OU completely unchanged. Consequently, we can ensure that all our inhibitory HUs do indeed have negative values for *w*_*j*_ and all excitatory HUs have positive ones by switching the signs of *w*_*j*_, *b*_*j*,_ and *w*_*jfτ*_ in all those HUs for which $\sum_{f,\tau }w_{jf\tau }$ was found to be negative after training. In the interest of easier interpretation, that is what we did, producing the 'partially adjusted' NRF model.

Next let us consider how to further adjust the network to have non-negative outputs. For the partially adjusted NRF model, threshold is simply the most negative output value -*ρ*_*1*_ (where weighted-output ranges from -*ρ*_1_*w*_j_ to +*ρ*_1_*w*_j_). The explanation for this is as follows: An excitatory HU (positive *w*_j_) can equivalently be seen as a neuron with a positive-only output, whose weighted-output goes from 0 to +2*ρ*_1_*w*_j_, acting on an OU whose resting state is less by -*ρ*_1_*w*_j_. An inhibitory HU (negative *w*_j_) can equivalently be seen as a neuron with a positive-only output, whose weighted-output goes from 0 to -2 *ρ*_1_*w*_j_, acting on an OU whose resting state is greater by +*ρ*_1_*w*_j_. Making these adjustments thus produces the final 'adjusted' network, which was used for all the results.

#### The inhibitory/excitatory score

As discussed, the HUs of the NRF model are not 'hard-wired' as excitatory or inhibitory, but each after fitting may nevertheless be “predominantly” excitatory or inhibitory in its influence on the OU. The sign of their output weight and the balance of positive and negative weights in the STRF will determine whether the HU is predominantly excitatory or inhibitory. To quantify the extent to which a HU is inhibitory or excitatory we calculated an inhibitory/excitatory (IE) score for each HU *j*:

$$IE=\text{sign}\left(w_{j}\right)\frac{\sum_{f,\tau }^{}w_{jf\tau }}{\sum_{f,\tau }^{}\left|w_{jf\tau }\right|}$$

This *IE* score is bounded between -1 and 1. For a positive *w*_*j*_, if all elements *w*_*jfτ*_ are non-negative (and at least one is not 0), IE = 1, if all elements are non-positive (and at least one is not 0), *IE* = -1. For a negative *w*_*j*_, the opposite is true. If the sum of the negative elements equals the sum of the positive elements, *IE* = 0. This measure was used to investigate whether excitatory or inhibitory HUs play different functional roles in the NRF (Fig 4E).

#### The expansive/compressive score

The distributions of HU activation in the nonlinearity plots (Fig 3B) are calculated with the adjusted model. The expansive/compressive (EC) score measures where the unit tends to operate along the nonlinear activation function:

$$EC=\frac{\rho_{6}}{\rho_{1}}\sum_{t}z_{j}\left(t\right)-\rho_{4}-\rho_{5}$$

The EC score is the average output of the unit over all the stimuli, scaled to be between -1 at threshold and +1 at saturation. For the NRF model *ρ*_1_ = 1.7159, *ρ*_*4*_ = 0, *ρ*_*5*_ = 0, *ρ*_*6*_ = 1 and for the OU we replace *z*_*j*_(*t*) with $\hat{y}\left(t\right)$. For the LN model, $\hat{y}\left(t\right)$ replaces *z*_*j*_(*t*), *ρ*_1_ and *ρ*_*4*_ are the fitted values from the nonlinearity, and *ρ*_*5*_ = 1 and *ρ*_*6*_ = 2. The average output (unscaled EC) for each unit is shown as the green dot on its nonlinear activation function (Fig 3A, 3B and 3D).

#### The displayed STRFs

The HU SRTF weights (Fig 3B, top panels of each HU) are shown with each element sign-reversed for the inhibitory HUs. Plotting HU STRFs in this manner thus ensures that the STRF plots always show the direction of effect of an STRF weight on the model output, rather than on the HU. This was done to facilitate the comparison between NRF HU STRFs and LN model STRFs.

#### Measuring the contours, and temporal and spectral tuning width of the STRFs

To obtain smooth contours for high sensitivity regions of the HU STRFs (Fig 3C), we spline interpolated the HU’s display STRF onto an evenly spaced grid at 8 times the resolution, with 7 additional values between each frequency, and 7 between each time. For the excitatory HUs the contours at half the maximum value of this matrix were plotted. For the inhibitory HUs, the contours at half the minimum value of this matrix were plotted.

To get a measure of the tuning width (Fig 4C and 4D), we calculated the power STRF by taking the square of each element of the interpolated STRF. Next, to measure the frequency tuning width, we summed the power STRF over all the time bins and then determined the maximum value of the resulting vector. The half-height frequency tuning width was defined as the number of elements of this vector ≥50% of this maximum value, multiplied by the frequency range covered by each bin in the interpolated STRF (⅙ × ⅛ = 1/48 octaves). Quarter-height frequency tuning widths were defined analogously at ≥25%. The half- and quarter-height temporal tuning widths were calculated analogously.
